# Physicochemical Properties and Efficacy of Poloxamer Bone Wax on Hemostasis at the Bone-Amputation Site

**DOI:** 10.34133/bmr.0191

**Published:** 2025-04-15

**Authors:** Dae Hyung Lee, Miri Kim, Yeji Choi, Mi Hee Lee, Jong-Chul Park

**Affiliations:** ^1^ Advanced Medical Device R&D Center, HansBiomed Co. Ltd., Seoul 05836, Republic of Korea.; ^2^ Cellbiocontrol Laboratory, Department of Medical Engineering, Yonsei University College of Medicine, Seoul 03722, Republic of Korea.

## Abstract

Bone wax, an essential material for bone hemostasis in orthopedic, thoracic, and neurological surgeries, is defined as a substance that physically controls bleeding caused by bone fractures. Absorbable bone-wax products such as poloxamer multiblock copolymers can be topically applied, form a physical barrier, are biocompatible, and can be absorbed by/excreted from the body. However, absorbable bone waxes continue to have limited physical properties, poor bone adhesion, and low hemostatic quality. When applied to the affected area, they quickly dissolve in blood and body fluids, preventing maintenance of the physical barrier over a certain period and thereby reducing the hemostatic effect. This study introduces a new type of absorbable bone wax (OSSTOP) constructed from 2 poloxamer multiblock copolymers with different molecular weight ranges. To determine whether OSSTOP overcomes the limitations of the existing products, the physicochemical properties and efficacy of OSSTOP were compared with those of 2 existing absorbable bone-wax products, OSTENE and NOVOSEAL. The adhesive strengths, yield loads, and solubilities of the products were evaluated and compared in vitro. Hemostasis at the bone-amputation site and absorption/degradation of the products were then evaluated through animal experiments in vivo. The biological safety (cytotoxicity) of the newly developed OSSTOP was also assessed. A histological analysis confirmed superior hemostasis at the bone-amputation site and a favorable biological response after treatment with OSSTOP. We expect that OSSTOP will improve the convenience, hemostatic performance, and biocompatibility of bleeding cessation at bone-amputation sites in the clinical environment.

## Introduction

As bones contain rich channels for blood and marrow, they are prone to local bleeding after surgical incision or traumatic fracture. Bleeding is especially severe around highly vascularized bones such as the spine and sternum. Bone wax, generally defined as a substance that physically controls bleeding caused by bone fractures, is an essential material for bone hemostasis in orthopedic, thoracic, and neurological surgeries. When physically compressed on a fractured or cut bone surface, bone-wax forms a physical barrier against bleeding [1–3].

Bone wax is divisible into 2 types with different characteristics: nonabsorbable and absorbable. The first bone-wax products described by Parker and Horsley in 1892 consisted of beeswax softened with isopropyl palmitate and paraffin wax. Beeswax is insoluble and cannot be metabolized or absorbed in the body; instead, it remains indefinitely at the application site, causing many complications. The most common complications are inhibited bone formation, infection, and foreign body reaction [3–9].

In 1950, Geary and Frantz combined Carbowax, poly(ethylene glycol) (PEG), and oxidized cellulose into an experimental absorbable bone wax. The literature from 1980 to 2000 reports numerous bone-wax substitute prototypes such as fatty acid salts, fibrin/collagen pastes, gelatin pastes, glycolic or lactic acid/glycerol oligomers, partially deacetylated chitin hydrochloride, PEG/microfibrillar collagen pastes, polydioxanone/natural oils, and polyorthoesters [4,5,10]. Unfortunately, none of these formulations have reached widespread use or entered the market, suggesting that the beneficial properties of traditional bone waxes are not easily combined with the advantages of absorbable materials.

In the 2000s, researchers developed an ideal absorbable bone-wax product that is easy to use and effective like traditional beeswax, but is completely absorbable, noninflammatory, and biocompatible. An absorbable product composed solely of alkylene oxide block copolymers was developed in 2001, followed by a commercially available water-soluble alkylene oxide copolymer-based bone wax in 2006. The commercial product is reportedly rendered smooth by manual manipulation, adheres well to bleeding bone, dissolves within 24 to 48 h at the grafted site, and is absorbed and excreted during the early stage of bone healing, allowing later bone formation [4,5,10].

Absorbable bone-wax products must be easily applied at the affected site, capable of forming a physical barrier, biocompatible, and readily absorbed by/excreted from the body. These requirements are satisfied by poloxamer multiblock copolymer [CAS No. 9003-11-6, synonyms pluronic, polyoxypropylene–polyoxyethylene block copolymer, PEG–PPG–PEG, PEO–PPO–PEO; PPG = poly(propylene glycol); PEO = poly(ethylene oxide); PPO = poly(propylene oxide)]. The length of the structurally repeating hydrophilic or hydrophobic blocks, sol–gel transition temperature, temperature range of existence as a gel, and the minimum gel-formation concentration in poloxamer multiblock copolymers are all controllable. The commercially available ABA triblock copolymer series of poloxamers, where A is a hydrophilic block (PEG or PEO) and B is a hydrophobic block (PPG or PPO), provides a pool of more than 50 amphiphilic, water-soluble, and polymorphic materials. The physical and chemical properties of the poloxamer copolymers can be finely tuned by modifying the molar mass ratio between the PEO and PPO blocks (from 1:9 to 8:2), potentially enabling direct modification of their properties and interactions with cells and cell membranes in vivo and the design of innovative nanomedicines and novel biomaterials. Physicochemical studies of aqueous poloxamer solutions have been recently reviewed [11,12]. In studies comparing the critical micelle concentrations at different overall compositions and temperatures, the conditions of amphiphilic ABA triblock copolymers lie between those of diblock (AB) polymers and BAB triblock copolymers with 2 hydrophobic sides [12]. As the temperature increases from 4 to 30 °C, a poloxamer aqueous solution transitions into a hydrogel as single molecules form micelles. Therefore, poloxamer hydrogel is composed of micelles packed into a certain structure, which easily erodes in the presence of excess water [13].

Absorbable bone-wax products are slowly dissolved in the body, absorbed, and excreted as they are transformed to the sol phase by body fluids at the body temperature. Grindel et al. [14,15] reported that approximately 85% of the poloxamer is excreted through the kidneys and feces within 48 h of application, and is completely excreted within 1 week.

The major bone-wax products on the current market are manufactured by Aesculap, CP Medical, Covidien, Ethicon, and Surgical Specialties. Nonabsorbable products causing side effects and complications are being replaced by absorbable products developed in the 2000s. A representative absorbable bone-wax product is OSTENE, which has been approved by the U.S. Food and Drug Administration. OSTENE is softened by manual manipulation before use and achieves hemostasis when applied to bleeding bones. Within 24 to 48 h, OSTENE dissolves into alkylene oxide copolymer, which is removed from the body without being metabolized. In addition, the copolymer is hydrophilic and well adheres to wet surfaces, promoting the hemostasis of bones. Therefore, OSTENE has a wide range of indications and is mainly applied to the bleeding area of bones during orthopedic surgery, thoracic surgery, and neurosurgery [5]. However, absorbable bone wax has certain limitations. When applied to a bleeding bone site, a substantial amount adheres to the user’s surgical gloves, making it difficult to apply the exact required amount, thereby causing inconvenience during use [16]. Additionally, during the hemostatic process, the bone wax tends to detach easily from the bleeding site or dissolve rapidly, leading to a loss of hemostatic effect. These drawbacks of bone wax have been reported in previous studies [5,17–19].

The efficacy of absorbable bone max products in reducing postoperative bleeding and transfusion rates has been evaluated in a clinical case study of total knee arthroplasty (TKA). The study compared the estimated blood losses, hemoglobin levels, and transfusion rates of patient groups receiving 2 types of hemostasis treatment after TKA: hemostasis with absorbable bone-wax products, and hemostasis with electrocautery. The absorbable bone-wax products safely and effectively reduced overall bleeding and blood loss, and patients receiving this treatment maintained higher hemoglobin levels than those in the electrocautery group [20,21].

The existing absorbable bone-wax products have been clinically used for a long time because they are low cost, are easily handled, are malleable, can be sutured, and readily adhere to bone. However, these products are disadvantaged by limited physical properties, poor bone adhesion, insufficient hemostatic quality, and interference with bone union, which have not been overcome. When applied to the affected area, they are quickly dissolved by blood and body fluids and cannot maintain a physical barrier over a sufficient period, which reduces their hemostatic effect.

Herein we develop a new type of absorbable bone-wax product called OSSTOP (HansBiomed Co. Ltd., Korea) by mixing and dissolving 2 poloxamer multiblock copolymers (PEG–PPG–PEG) with different molecular weight ranges. Through stability tests and animal studies, we compare the physical and chemical properties, biological safeties, and efficacies of OSSTOP and the existing absorbable bone-wax products, verifying that the new product can improve the limitations of the existing products.

## Materials and Methods

### Preparation of the test materials

The absorbable bone-wax product OSSTOP (HansBiomed Co. Ltd., Korea) was produced by mixing and dissolving 2 poloxamer multiblock copolymers with different molecular weight ranges. The liquid poloxamer [poly(ethylene glycol-ran-propylene glycol), 438200, Sigma-Aldrich, USA] and the powder poloxamer (Kolliphor P 338 Geismar, 50424591, BASF Corporation, USA) were mixed at a certain ratio and then dissolved to synthesize the product. The thermal synthesis method is detailed in “Biocompatible composition for bone hemostasis material, treatment method using same, and method for preparing same” (Korean Patent No. 10-2133503) and is only briefly described here. The liquid and powder poloxamers were mixed at a certain ratio, dissolved by heating at 80 °C for 30 min, and then poured into a mold for molding and curing at room temperature (Table 1). As the reference products for comparison, we selected OSTENE (Baxter Healthcare Corporation, United Kingdom) and NOVOSEAL (CG Bio Co. Ltd., Korea), whose raw materials are composed of poloxamer multiblock copolymers.

**Table 1.** Curing status (block formation) based on liquid and powder poloxamer mixing and dissolution conditions

	PEG-ran-PPG^a^ (%)		P 338^b^ (%)	Melting conditions	Curing conditions	Curing status
Mixing ratio	Group A	15	85	80 °C/30 min	Room temperature (15–25 °C)	Solid block
	Group B	25	75			Solid block
	Group C	35	65			Solid block
	Group D	45	55			Solid block
	Group E	55	45			Solid block
	Group F	65	35			Solid block (sticky)
	Group G	75	25			Solid block (very sticky)
	Group H	85	15			Solid block (very sticky)

^a^ PEG-ran-PPG:poly(ethylene glycol-ran-propylene glycol)/liquid poloxamer.

^b^ P 338: Kolliphor P 338 Geismar/powder poloxamer.

### Physical properties under the mixing and dissolving conditions of the poloxamer

Various solid blocks were formed by mixing and dissolving the liquid and powder poloxamers at ratios of 15 to 85 wt %. To measure the minimum force on the dough after forming the solid block, the sample was placed on the 3-axis bending jig of a tensile tester (LLOYD’s Long travel extensometer). The maximum force (yield load) was measured at the point of bending or breakage of the sample under a vertical loading of 5 mm/min supplied by the upper jig. The adhesive surface force for physical hemostasis was measured on 1 g of the material, which was removed from the solid block and kneaded into a sphere, and then placed between the grips of the compression/tensile tester under a constant load (50 ± 5 N). The maximum force at the moment of falling was measured while the 2 grips were moved at 50 mm/min.

### Analysis of molecular weight distributions and thermal properties of the poloxamers before and after synthesis

To confirm the stabilities of the liquid and powder poloxamers during thermal synthesis, the changes in molecular weight distributions and thermal properties of the poloxamers after synthesis were measured using gel permeation chromatography (GPC) and differential scanning calorimetry (DSC), respectively. For molecular weight analysis [EcoSEC HLC-8420 GPC (RI-Detector), Tosoh], the sample was completely dissolved in tetrahydrofuran and injected at 3 mg/ml. The thermal property changes (DSC 204 F1 Phoenix, NETZSCH) were measured while heating the samples from 30 to 300 °C at 10 °C/min.

### Comparative physical property analysis of the proposed and commercialized bone-wax products

To analyze the physical characteristics of the absorbable bone-wax products, we compared their adhesive strengths, yield loads, and solubilities within the environment of clinically indicated absorbable bone-wax products. In particular, we measured the adhesive strength of each product to the bone surface, required force of kneading the material before application, and time of dissolution through reactions with body fluids. The adhesive strength and yield load of each material were measured as described in the “Physical properties under the mixing and dissolving conditions of the poloxamer” section. For the solubility test, 2 g of each test substance was kneaded into a sphere, immersed in 10 ml of phosphate-buffered saline (PBS), and stored in a 37 °C incubator. The dissolution patterns and weight changes were recorded over time at the hourly intervals of 5 h. A control experiment using fetal bovine serum (FBS) was conducted (Fig. S1), as it better simulates physiological conditions

### Cytotoxicity evaluation of the selected bone-wax products

**Fig. 1.**
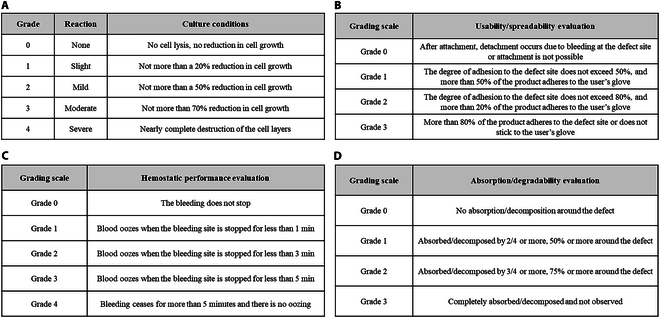
Grade classification per evaluation. (A) Cytotoxicity grades of bone-wax products. (B) Usability/spreadability grades. (C) Hemostatic performance grades. (D) Absorption/degradability grades.

The cytotoxicity of the bone-wax product was quantified following the ISO 10993-5 and ISO 10993-12 standards. Briefly, OSSTOP was extracted in polar (sodium chloride) and nonpolar (dimethyl sulfoxide) elution solvents at 37 ± 1 °C for 72 ± 2 h. The elution rate was 0.2 g/ml. Next, monolayers of L-929 mouse fibroblast cells were dosed with OSSTOP extracts eluted with polar or nonpolar solvents treated at 10% or 0.5% of the culture medium volume, respectively, and incubated at 37 ± 1 °C, 5% ± 1% CO_2_, 95% humidity for 48 ± 2 h. The incubated cells were observed under an optical microscope. Finally, each well of the OSSTOP extract, blank test solution, negative control solution, and positive control solution was observed under a microscope and graded as specified in Fig. 1A.

### Evaluation at the bleeding site after bone amputation in rats

The usability, spreadability, hemostasis performance, and hemostasis maintenance of each test substance were evaluated at the bleeding site of an amputated bone [16,22–25]. Animal experiments, performed on Sprague–Dawley (SD) male rats over 8 weeks of age, complied with the “Animal Protection Act” and the “Act on Laboratory Animals” of the Republic of Korea, and with the animal experimental methods of the Animal Experiment Ethics Committee of Konkuk University (Animal Experiment Approval Number: KU19201). Rats were respiratory anesthetized with isoflurane (oxygen flowmeter, 0.9 l/min; 3.5% isoflurane with 100% O_2_) in an induction chamber. After anesthesia induction, isoflurane anesthesia was maintained through a mask (oxygen flowmeter, 0.4 to 0.8 l/min; 1.5% isoflurane with 100% O_2_). Throughout the test period, the core body temperature was maintained at 37 to 38 °C using a warming mat. The skin and muscles of the femoral area of ​​the rat were incised to expose the bone, and the circular defect site (one site/femur) was marked. While injecting and aspirating saline into the exposed area, a 2-mm circular defect graft site was prepared through intermittent use of a hand drill. The circular defect was consistently formed at the diaphysis adjacent to the gluteal tuberosity. Each test substance (10 mg, kneaded in advance) was evenly filled or spread onto the circular defect area using sterilized surgical gloves or surgical tools. The experimental group was OSSTOP, and the comparative groups were commercially available OSTENE and NOVOSEAL bone waxes.

#### Usability/spreadability evaluation

The tissue adhesion, spreadability, and application ease of each hemostatic agent and the untreated control group were graded as shown in Fig. 1B.

#### Hemostatic performance evaluation

The hemostatic performance of each test group and the untreated control group was evaluated for 5 min and graded as shown in Fig. 1C.

#### Evaluation of hemostasis maintenance

After evaluating the usability/spreadability and hemostasis performance, the hemostasis maintenance of each test substance was evaluated during six 10-min intervals (total evaluation time, 1 h). The times of blood oozing were recorded, and the bleeding amounts were measured from the blood contents absorbed on preweighted paper.

### Evaluation of absorption/degradability after transplant at the bone-amputation bleeding site

The absorption/degradability extent and pattern of each test substance in the body were evaluated after implanting the test substance at the hemorrhage sites of amputated bones [16,22–26] of 10-week-old SD male rats. These experiments complied with the “Animal Protection Act” and the “Act on Laboratory Animals” of the Republic of Korea and with the Animal Experiment Ethics Committee of Konkuk University (Animal Experiment Approval Number: KU21007). Prior to implanting the test substance into the femur, the animals were prepared as described in the “Evaluation at the bleeding site after bone amputation in rats” section. Each test substance (50 g, kneaded into circular defects in advance) was evenly filled or spread over the circular defect site using sterilized surgical gloves or surgical tools and then attached. For absorption/degradability evaluations, the incised muscles and skin of the implantation site were sutured to retain the sample. The transplant site was then observed at 1, 3, 7, and 14 d. At the end of the observation period, the animals were euthanized, and the degree of absorption/decomposition and biological response of the transplant site were evaluated. The surrounding tissues were removed to evaluate the degree of inflammation. The experimental and comparison groups were treated with OSSTOP and commercialized OSTENE, respectively, and the untreated control group was also evaluated. The absorption/degradability grades are listed in Fig. 1D.

For long-term observation, local tissue irritation at the implantation site was evaluated by implanting the test materials into the femurs of healthy New Zealand White rabbits (weighing over 3 kg) for 13 weeks. Each group consisted of 8 animals (4 males and 4 females). This study was approved by the Animal Ethics Committee of the Korea Testing & Research Institute Healthcare Research Center in accordance with the “Animal Protection Act” and the “Act on Laboratory Animals” of the Republic of Korea.

For each rabbit, the test and control materials were implanted at 3 sites, spaced at least 2 cm apart, with approximately 0.1 g per site. After implantation, the surgical sites were sutured appropriately, and general symptoms were monitored until the end of the implantation period.

For hematological analysis, the animals were fasted overnight prior to blood collection. They were anesthetized with isoflurane, and approximately 2 ml of blood was collected from the abdominal aorta. The blood samples were placed in EDTA tubes (K2 EDTA 3.6 mg, BD Vacutainer, USA) and analyzed using a hematology analyzer (ADVIA 2120i, SIEMENS, USA) for various parameters. Blood coagulation time was measured using plasma obtained by centrifuging blood samples collected in 9NC Citrate tubes (Buffered Sodium Citrate 0.109M, 3.2%, BD Vacutainer, USA) at 3,000 rpm for 10 min. The coagulation time was analyzed using a blood coagulation analyzer (ACL ELITE PRO, Instrumentation Laboratory, USA).

At the end of the study, macroscopic evaluation of the implantation sites was performed, and the implant areas were excised for histological analysis. The experimental and control groups were treated with OSSTOP and the commercially available OSTENE, respectively. Changes in body weight during the implantation period are presented in Fig. S2.

### Histopathological evaluation

For histopathological analysis, the femurs and surrounding tissues were fixed in 10% neutral buffered formalin and decalcified using Solution Lite (Sigma-Aldrich) prior to routine tissue processing. Each specimen was then embedded in paraffin, and 4-μm-thick tissue sections obtained through the grafted area were stained with hematoxylin and eosin. The types and degrees of inflammatory responses and bone regeneration at the cortical bone defect sites in the control, comparative, and experimental groups were compared according to international standard guidelines (ISO 10993-6) at each time point.

At the end of the long-term observation study, surviving rabbits were anesthetized with isoflurane for blood collection and humane euthanasia. A macroscopic examination was conducted on the external surface, all orifices, the cranial cavity, and all thoracic and abdominal organs, including their contents.

Following the necropsy, a macroscopic evaluation was performed for all animals, and the weights of the heart, liver, lungs, spleen, kidneys, adrenal glands, testis, epididymis, ovaries, brain, pituitary gland, thymus, and uterus were measured. The organ weight measurements and necropsy findings are presented in Table S1, while the results of the hematological and blood biochemical analyses are provided in Table S2.

### Statistical analysis

Statistical analyses were conducted using Prism 4.02 (GraphPad Software, San Diego, CA, USA). Significant differences between groups were assessed using one-way analysis of variance. Multiple comparisons of the parametric data were then determined using Bonferroni’s post hoc test. In all tests, a *P* value of 0.05 was considered as statistically significant.

## Results

### Physical properties under the mixing and dissolution conditions of the poloxamer

**Fig. 2.**
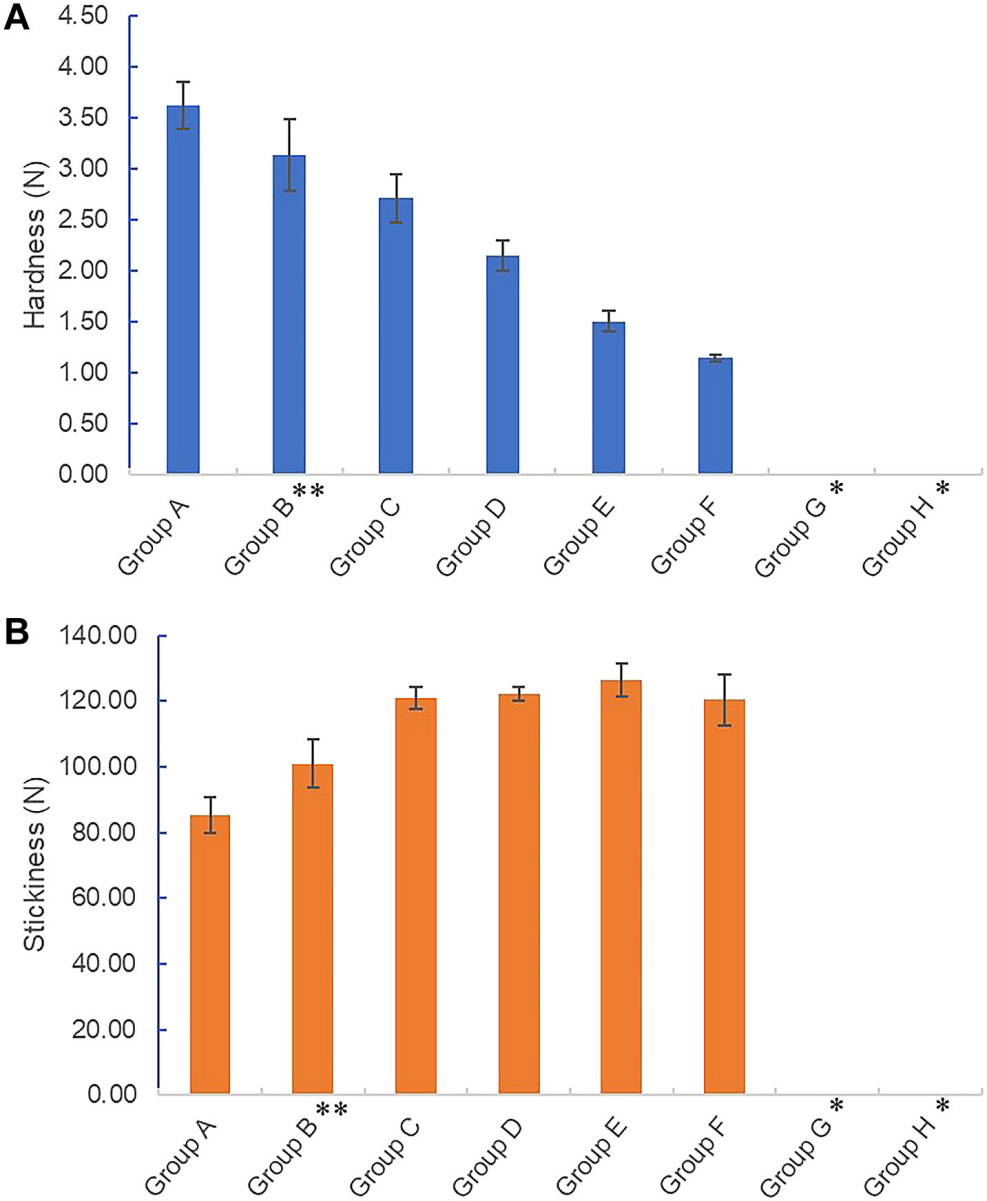
Confirmation of physical properties (i.e., hardness and stickiness) of liquid and powder poloxamer under different mixing and dissolution conditions. Liquid and powder poloxamer were mixed and dissolved based on the conditions described in the next. For each sample, 1 g was taken, kneaded into a spherical shape 10 to 20 times, and then measured. (A) Substance hardness. (B) Substance stickiness. *Groups G and H: not measurable. **Group B: (PEG-ran-PPG:P 338 = 25%:75%) = Selected bone wax = OSSTOP.

Liquid poloxamer [poly(ethylene glycol-ran-propylene glycol), 438200, Sigma-Aldrich, USA] and powder poloxamer (Kolliphor P 338 Geismar, 50424591, BASF Corporation, USA) were mixed at ratios of 15 to 85 wt % (Table 1) and then dissolved and cured to prepare the mixture. The degree of synthesis was observed during curing at room temperature. When the mixing ratio of the liquid poloxamer reached or exceeded 65%, block formation was difficult and the product became viscous. Solid-block poloxamers were formed at mixing ratios of 15% to 55% for the liquid poloxamer and 15% to 55% for the powder poloxamer. Solid blocks prepared at these mixing ratios maintained their usability and adhesiveness after kneading and spreading. In addition, the hardness and stickiness of the synthesized poloxamers depended on the mixing ratios of the liquid and powder poloxamers (Fig. 2). Increasing the mixing ratio of the liquid poloxamer decreased the hardness of the poloxamer in the solid-block state after synthesis (Fig. 2A). Meanwhile, the stickiness increased as the ratio increased to more than 35% (Fig. 2B). Among the viable mixing ratios of the liquid and powder poloxamers, we selected the condition yielding similar physical properties to those of commercialized bone wax Group B (PEG-ran-PPG:P 338 = 25%:75%).

### Molecular weight distributions and thermal properties of the poloxamers before and after synthesis

**Fig. 3.**
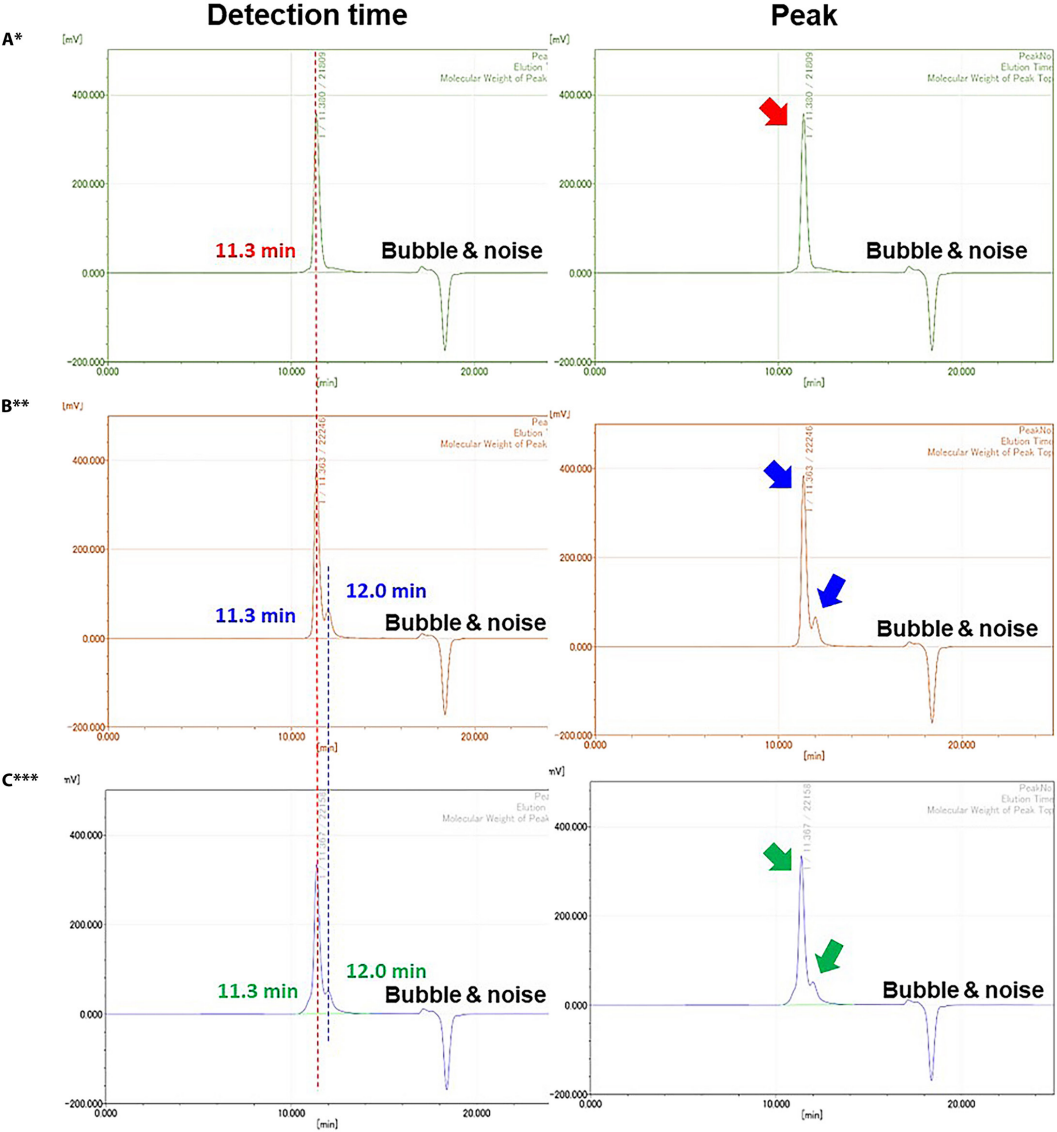
Molecular weight distributions of liquid and powder poloxamer under different synthesis conditions. (A) Native molecular weight peak of PEG-ran-PPG (red). (B) Native molecular weight peak of P 338 (blue). (C) Native molecular weight peak of OSSTOP (green). All panels reflect respective synthesis conditions. *PEG-ran-PPG: Poly(ethylene glycol-ran-propylene glycol)/liquid poloxamer. **P 338:Kolliphor P 338 Geismar/powder poloxamer. ***OSSTOP = Selected bone wax = Group B (PEG-ran-PPG:P 338 = 25%:75%).

**Fig. 4.**
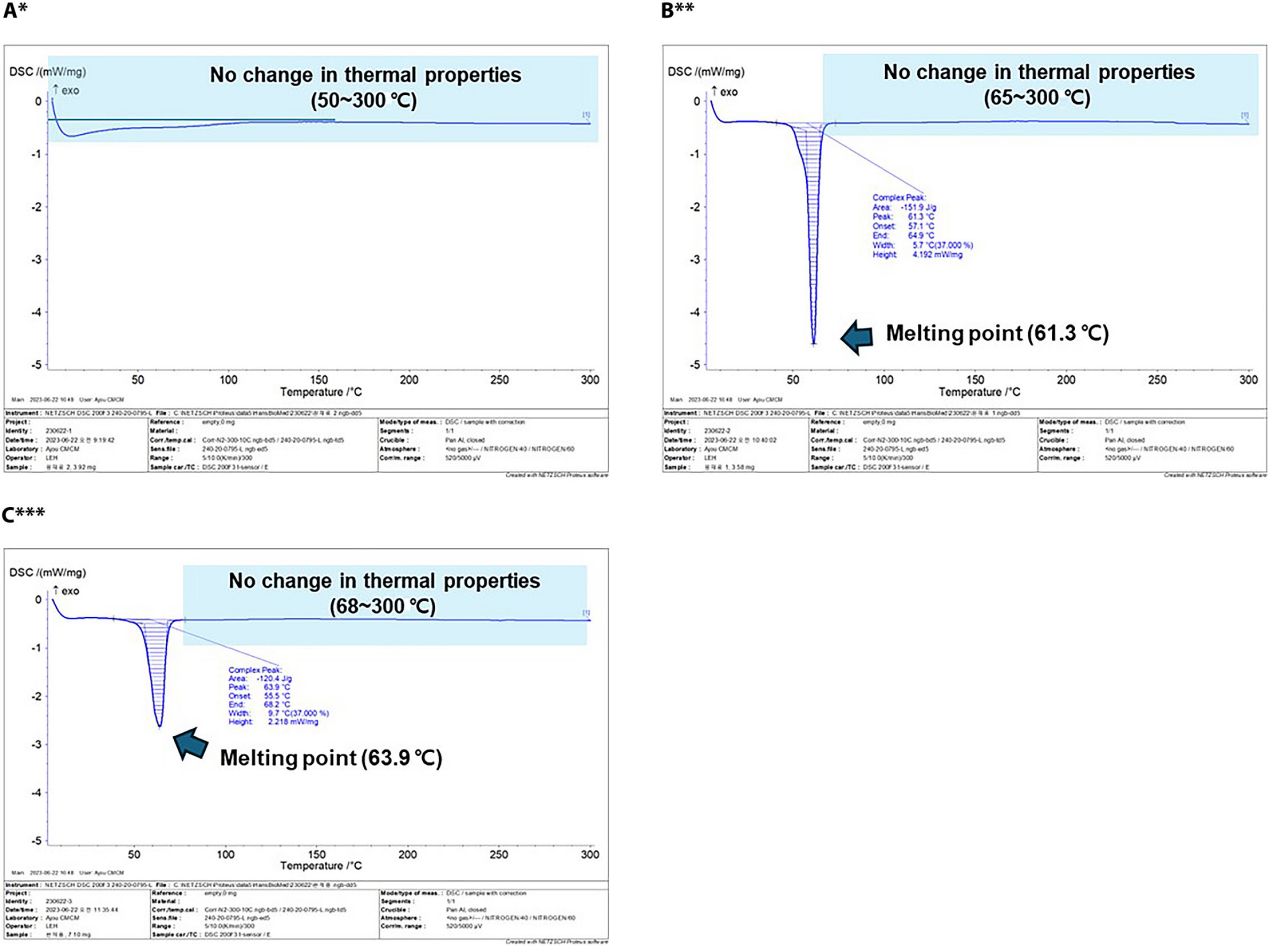
Changes in thermal properties of liquid and powder poloxamer under different synthesis conditions. (A) PEG-ran-PPG. (B) P 338. (C) OSSTOP. All panels reflect respective synthesis conditions. *PEG-ran-PPG: Poly(ethylene glycol-ran-propylene glycol)/liquid poloxamer. **P 338: Kolliphor P 338 Geismar/powder poloxamer. ***OSSTOP = Selected bone wax = Group B (PEG-ran-PPG:P 338 = 25%:75%).

To confirm the unique characteristic changes afforded by the 80 °C dissolution process under the selected synthesis conditions of Group B (PEG-ran-PPG:P 338 = 25%:75%), we determined the molecular weight distributions and thermal properties of the poloaxamers in OSSTOP (Figs. 3 and 4). Prior to mixing, the unique molecular weight distributions of the liquid and powder poloxamers were confirmed from the unique molecular weight peaks in GPC and DSC. The spectrum of the liquid poloxamer showed a single peak at 11.3 min (Fig. 3A, red arrow), whereas that of the powder poloxamer showed 2 peaks at 11.3 and 12.0 min (Fig. 3B, blue arrows). The above peaks were also detected in OSSTOP (Fig. 3C, green arrows), confirming a stable thermal synthesis with no change in molecular weight. In addition, the heat-induced changes in the characteristics of the liquid poloxamer, powder poloxamer, and OSSTOP during synthesis were evaluated in the temperature ranges 30 to 55 °C, 57 to 65 °C, and 55 to 68 °C, respectively. All samples were endothermic, and their thermal behaviors confirmed a melting point beyond which the thermal flow was constant with no changes in thermal characteristics (68 to 300 °C; Fig. 4).

### Physical properties (adhesion, yield load, solubility) of OSSTOP and commercialized bone wax

**Fig. 5.**
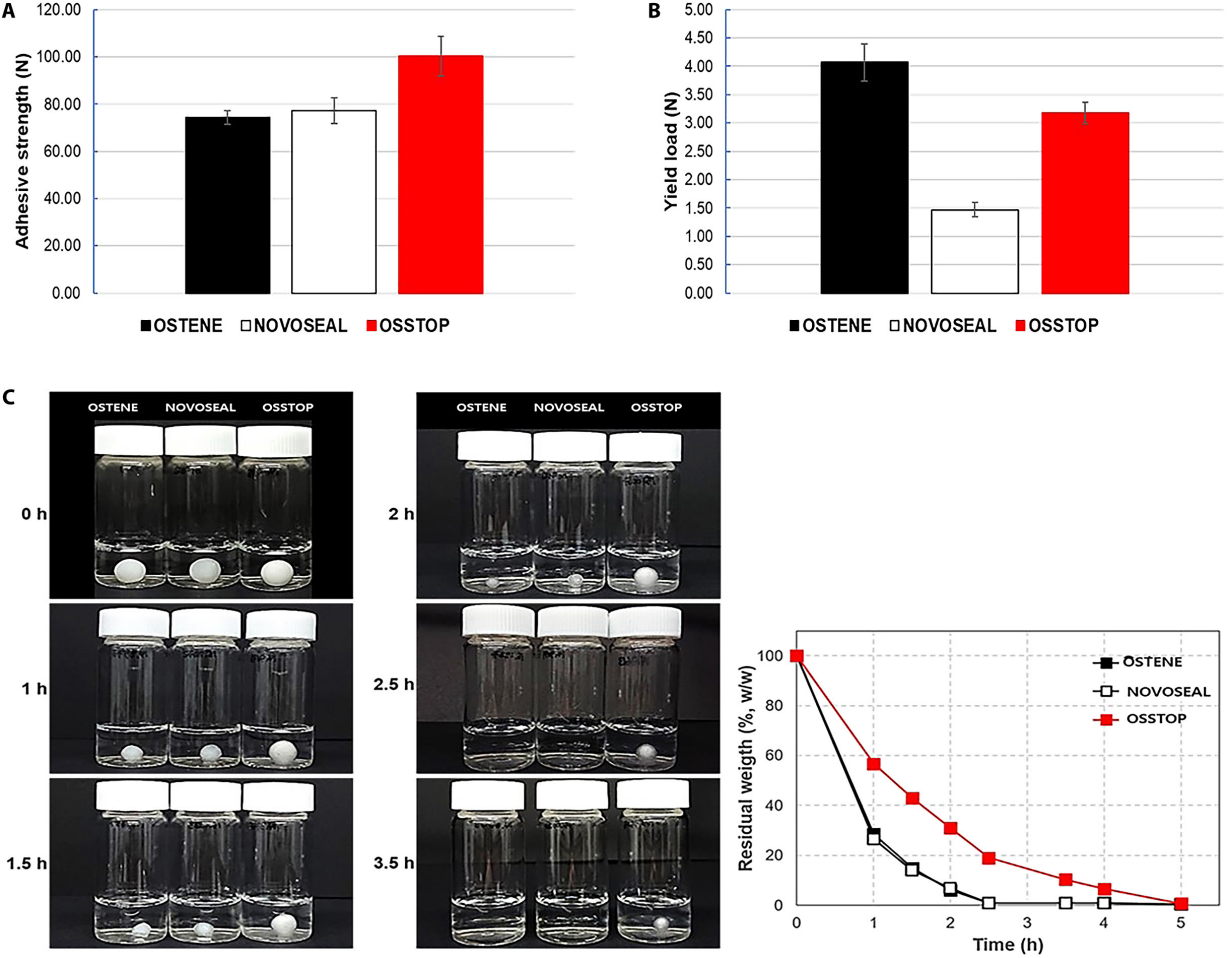
Physical property analysis of OSSTOP and commercialized bone-wax products. (A) Adhesion comparison of bone wax. (B) Yield load comparison of bone wax. (C) Solubility comparison of bone wax.

Considering the clinical use environment of OSSTOP and the commercialized OSTENE and NOVOSEAL products, we evaluated and compared their adhesive strengths to bone surface (adhesion), required kneading forces before application in the body (yield loads), and dissolution times when reacting with body fluids (solubilities). Under the same conditions, the adhesive strengths of OSTENE and NOVOSEAL were 74.25 ± 2.92 N and 77.24 ± 5.38 N, respectively. In OSSTOP, the adhesive strength was raised to 100.24 ± 8.27 N (Fig. 5A). Meanwhile, the yield loads of OSTENE, NOVOSEAL, and OSSTOP were 4.07 ± 0.33 N, 1.47 ± 0.12 N, and 3.17 ± 0.19 N, respectively (Fig. 5B). Finally, the solubility measurements confirmed similar dissolution patterns and weight changes in OSTENE and NOVOSEAL over time, and a later dissolution time of OSSTOP than of the commercialized bone waxes (Fig. 5C). Figure S1 shows a similar solubility when treated with PBS and FBS.

### Cytotoxicity of OSSTOP

The test results of the negative and positive control groups satisfied the validity criteria, confirming that the cytotoxicity test was properly conducted. In addition, the test substance caused no separation of granules in the cytoplasm of the cultured cells, cell lysis, or cell-growth inhibition under any condition. Therefore, the qualitative morphological reactivities of the cells exposed to the test substance, whether eluted with a polar solvent or with a nonpolar solvent, were judged as “None”. In summary, the cytotoxicity response grade of OSSTOP under the test conditions was judged as “Grade 0” (Table 2).

**Table 2.** Cytotoxicity tests assessing the biocompatibility of OSSTOP

Extract (test substance)								Control substance										
Polar solvent				Nonpolar solvent				Blank test				Negative control				Positive control		
0	0	0		0	0	0		0	0	0		0	0	0		4	4	4

### Evaluation of the bone excision bleeding area

#### Usability/spreadability results

**Fig. 6.**
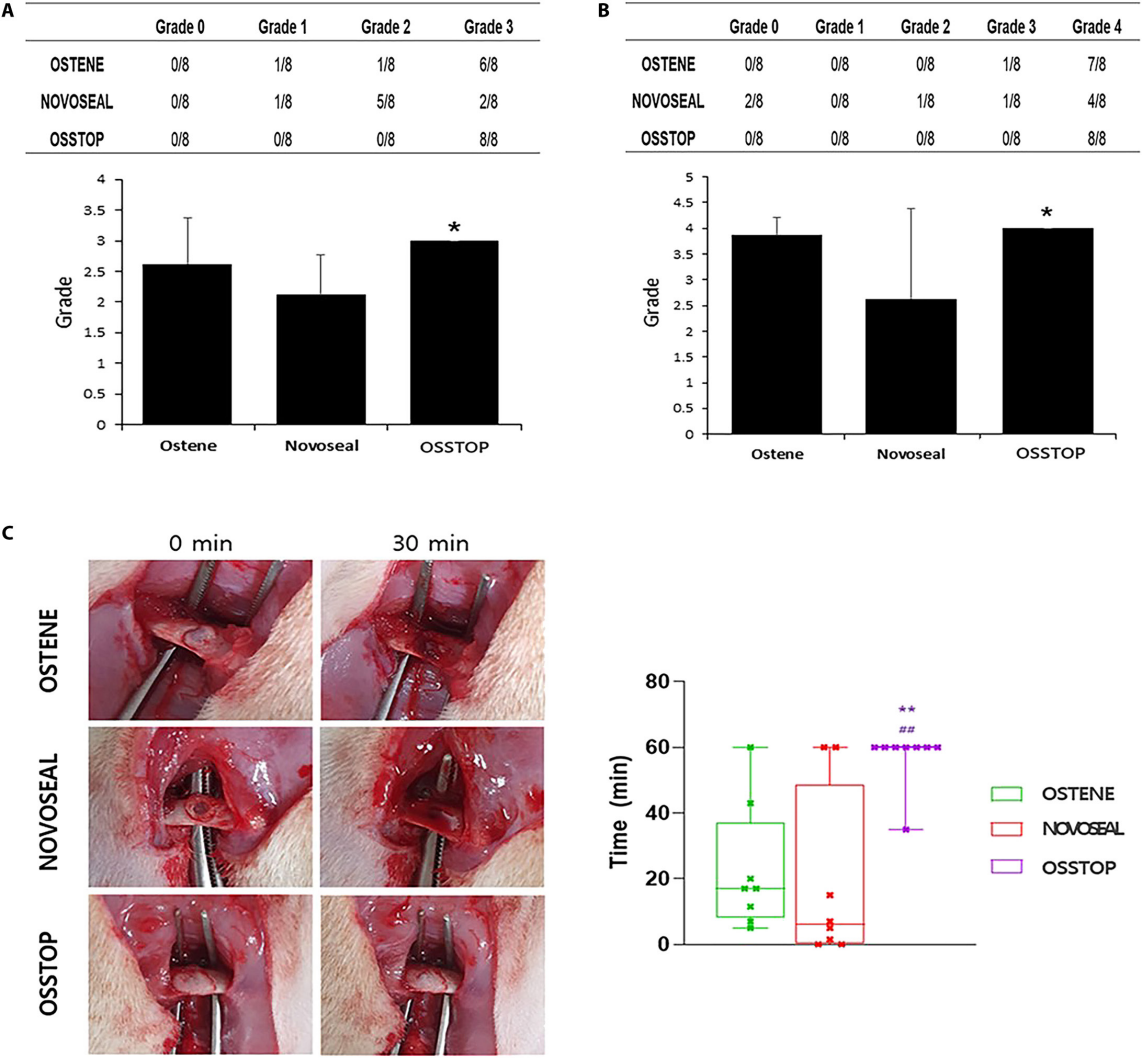
Evaluation of bone excision bleeding area. (A) Results of bone-wax usability/spreadability evaluations as determined via animal experiment (*P < 0.05 compared to NOVOSEAL). (B) Results of bone-wax hemostatic evaluations as determined by animal experimentation (*P < 0.05 compared to NOVOSEAL). (C) Results of bone-wax hemostasis maintenance evaluations as determined by animal experimentation; chart depicting hemostasis maintenance patterns over time after bone-wax application, i.e., the results of hemostasis maintenance over time following bone-wax application (*P < 0.05; **P < 0.01, compared to NOVOSEAL; ^#^*P* < 0.05, compared to OSTENE).

No specimens were judged as “Grade 0” during the usability/spreadability evaluations of OSTENE, NOVOSEAL, and OSSTOP. In the NOVOSEAL evaluation, 5 of 8 specimens were judged as “Grade 2” and the OSTENE evaluation yielded 1 “Grade 1” specimen, 1 “Grade 2” specimen, and 6 “Grade 3” specimens. In the OSSTOP evaluation, all 8 specimens were judged as “Grade 3”. Most of the NOVOSEAL bone wax was adhered to the glove, limiting the molding and quantitative application of this product, but OSSTOP never adhered to the glove, enabling easy molding and quantitative attachment in all specimens (Fig. 6A).

#### Hemostatic performances of the bone waxes

Hemostatic performance was evaluated based on the presence and timing of oozing during the 5-min period after applying the bone wax. After application of NOVOSEAL, 4 of 8 specimens were observed as “Grade 4” and the remaining specimens exhibited various degrees of hemostatic performance (‘Grade 0” to “Grade 3”). “Grade 4” was achieved by almost all specimens (7/8) in the test group OSTENE and by all 8 specimens in the test group OSSTOP. The hemostatic performance of the test group NOVOSEAL was low in specimens with low usability/spreadability evaluation grades, whereas the hemostatic performance of OSSTOP was similar to that of the control group OSTENE (Fig. 6B).

#### Hemostasis maintenance abilities of the bone waxes

To measure the hemostatic maintenance time, we recorded the bleeding time for 1 h after applying the bone wax. The average hemostasis maintenance times of NOVOSEAL and OSTENE were 18.6 ± 26.0 min and 22.5 ± 19.1 min, respectively. In the test group OSSTOP, the hemostasis state was maintained for 56.8 ± 8.8 min. The NOVOSEAL bone wax was detached from the defect site after oozing, whereas OSTENE and OSSTOP remained within the defect site for a considerable time following oozing (Fig. 6C).

### Absorption/degradability abilities of the bone waxes after transplantation at the bone-amputation bleeding site

**Fig. 7.**
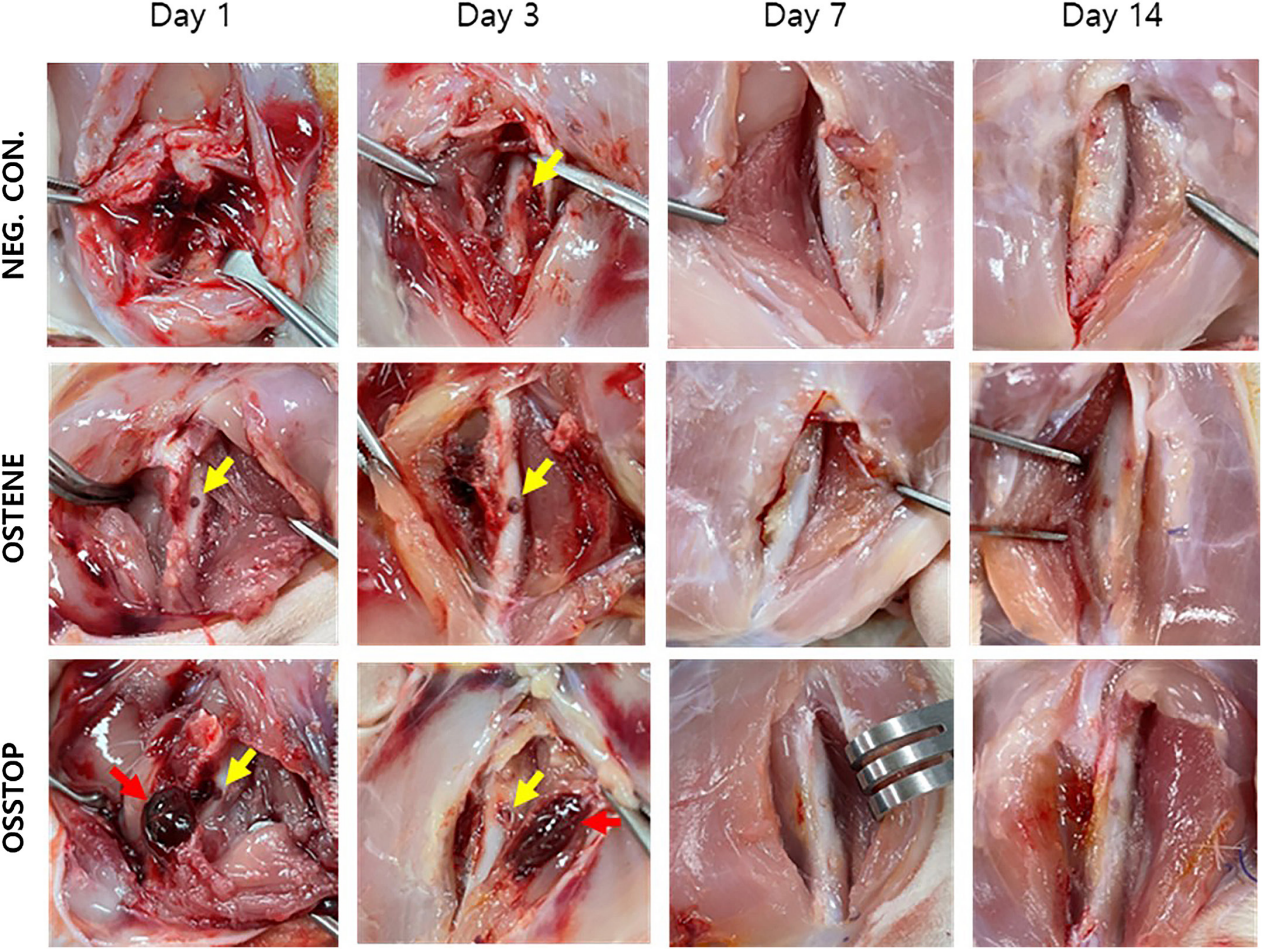
In vivo absorption/degradation in a bone-wax animal transplantation model. Autopsies were performed 1, 3, 7, and 14 d after each application of bone wax, at which time the application site was visually evaluated. On days 1 and 3 after bone-wax application, only a small amount of bone wax was observed within the defect (yellow arrow); moreover, in the OSSTOP application group, we observed coagulated blood clots that were not adhered to surrounding tissue (red arrow); 7 d after the application of bone wax, the defect disappeared in all groups because of granulation tissue or bone regeneration within the area of the bone defect.

The bone-wax application sites were visually evaluated following autopsy at 1, 3, 7, and 14 d. Trace amounts of OSTENE and OSSTOP remained in the defect at 1 d post-application (Fig. 7, yellow arrows), scoring “Grade 2”. Whereas the OSTENE group showed little or no test material in the surrounding tissues, OSSTOP showed a coagulated blood clot (Fig. 7, red arrow) above the defect without adhesion to the surrounding tissues. The blood clot remained until 3 d post-application of OSSTOP but was absorbed to nonvisible levels by day 7. On days 7 and 14 after bone-wax application, the bone defects in all groups were removed by granulation tissue or bone regeneration (Fig. 7).

### Histopathological evaluation

**Fig. 8.**
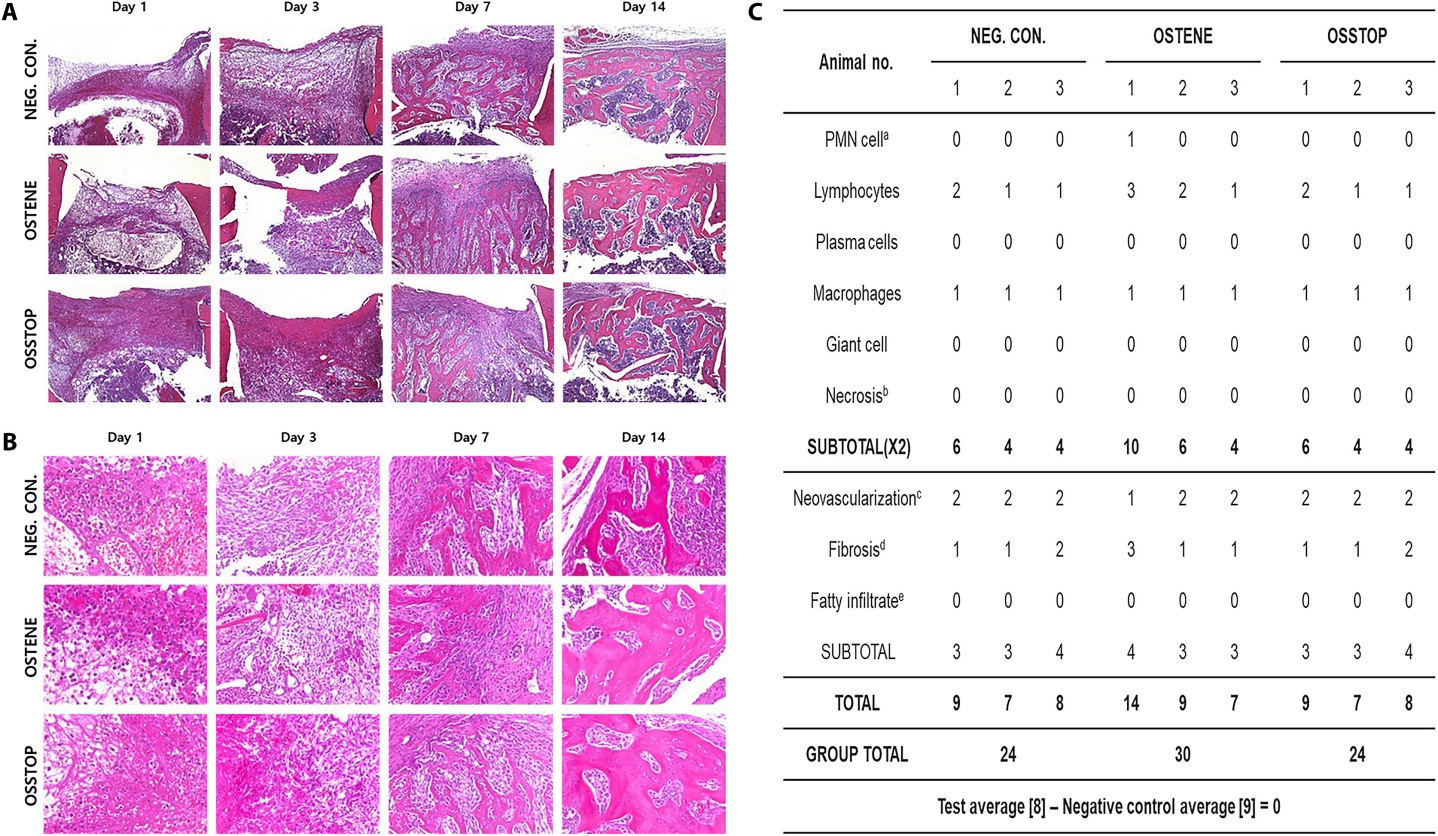
Histopathological evaluations. (A) Cortical bone defects in a bone-wax animal transplantation model. (B) Inflammation and bone regeneration in cortical bone defects in a bone-wax animal transplant model. (C) Inflammation and bone regeneration in cortical bone defects 14 d after bone-wax animal transplantation. a: 0, none; 1, rare; 2, 5 to 10/high-power field (hpf); 3, heavy infiltrate; 4, packed. b: 0, none; 1, minimal; 2, mild; 3, moderate; 4, severe. c: 0, none; 1, minimal capillary proliferation; 2, groups of 4 to 7 capillaries with supporting fibroblastic structures; 3, broad band of capillaries with supporting structures; 4, extensive band of capillaries with supporting structures. d: 0, none; 1, narrow band; 2, moderately thick band; 3, thick band; 4, extensive band. e: 0, none; 1, minimal amount of fat associated with fibrosis; 2, several layers of fat and fibrosis; 3, elongated and broad accumulation of fat cells about the implant site; 4, extensive fat completely surrounding the implant.

The types and degrees of inflammatory response to cortical bone defects were compared between the control group and the comparison and experimental groups at each autopsy time point. According to the international standard guideline (ISO 10993-6), the OSSTOP test group was evaluated at 8 points (lower or similar to the number of evaluation points on the control group; 9 points) 14 d after application. The tissue-regeneration responses did not significantly differ among the OSSTOP group, test group, control group (Neg. Con.), and comparison group (OSTENE), indicating that OSSTOP does not inhibit bone regeneration (Fig. 8).

In the long-term observation study of femoral implantation in the rabbit model, no animal deaths occurred in any test group throughout the study period. Additionally, no abnormal symptoms related to the implantation of the test materials were observed in any group, including the control group.

There were no statistically significant changes in body weight or necropsy findings. While some statistically significant differences (*P* < 0.05) were observed in organ weights, hematological analysis, and blood biochemical tests compared to the control group, all measured values remained within the normal range for both male and female rabbits. Therefore, these changes were not considered toxicological effects related to the implantation of the test material (Fig. S2 and Table S2).

Macroscopic examination of the implantation site and surrounding tissues revealed no notable lesions related to the test material. In the histological evaluation, the scores for both male and female rabbits were 0.0, indicating no adverse tissue reactions (Table S1). Furthermore, no abnormalities or test material-induced lesions were observed in the major organs of either the control or experimental groups.

## Discussion

To alleviate the shortcomings of the existing commercial absorbable bone waxes, we developed an absorbable bone wax with improved usability and physical properties from liquid and powder poloxamers. Unlike existing bone-wax products, a new type of absorbable bone wax was developed by combining 2 poloxamer multiblock copolymers (liquid poloxamer and powder poloxamer) with different molecular weight ranges and physical states through thermal compatibility. The bone wax developed in this study has a higher molecular weight than the poloxamer multiblock copolymers used in existing bone-wax products, and its stability was confirmed through GPC and DSC analyses based on thermal compatibility. By modifying the molecular weight range of the raw materials and the thermal compatibility method, the hydrophobic block content of PPG (PPO) was increased, significantly improving the previously weak physical properties of existing bone-wax products, such as adhesion, yield load, and solubility.

Animal experiments and clinical trials have confirmed the many advantages of absorbable bone wax over nonabsorbable bone wax. Because absorbable bone wax is dissolved after hemostasis of the bone-amputation site, bone regeneration is uninhibited and the risk of bone nonunion and infection is significantly reduced [16,22–25]. Among numerous raw materials considered for absorbable bone wax, absorbable materials such as oxidized regenerated cellulose, microfibrillar collagen, and gelatin are best absorbed at the application site during in vivo animal experiments. Oxidized regenerated cellulose is eliminated from the application site within 6 weeks to 1 year, and microfibrillar collagen and gelatin are absorbed within 14 to 90 d [3]. The decomposition period of the recently commercialized absorbable bone wax based on an alkylene oxide copolymer is considerably shortened to 48 to 72 h. In addition, the degraded/absorbed poloxamer is eliminated through renal or hepatic excretion [25,27].

However, when utilized in clinical environments, the usability of poloxamer-based absorbable bone wax is limited by the physical properties of the bone wax. The low mechanical strength and rapid dissolution of the poloxamer in the body impedes hemostasis [17–19]. In addition, the poloxamer-based bone mass has a poor molding texture and attaches to surgical gloves during kneading [16].

By thermally synthesizing 2 types of poloxamer, we intended to improve the molding texture and decomposition period of absorbable bone wax. First, we adjusted the hardness of the developed OSSTOP to a value similar to those of commercially available absorbable bone waxes, minimizing the force required for kneading in a clinical environment. To improve the molding texture, we changed the content ratio of the hydrophobic block PPG (PPO) using liquid and powder poloxamers. In their review of amphiphilic ABA triblock copolymers, Booth and Attwood [12] reported that the critical micelle concentration can be controlled by adjusting the type, position, and length of the B (hydrophobic) block. Overall, the critical micelle concentration increases by increasing the length of the B block [12]. The developed OSSTOP was thermally synthesized from a liquid poloxamer and a powder poloxamer. The increased length of the B blocks raised the melting point of the product from that of the powder poloxamer, thereby increasing the adhesion and solubility of OSSTOP while retaining the hardness of the poloxamer. That is, thermally synthesizing the liquid and powder poloxamers increases the overall critical micelle concentration from that of a single poloxamer. In conclusion, one can judge that thermal synthesis changed the content of the hydrophobic block PPG (PPO) and increased the overall critical micelle concentration, hence increasing the adhesiveness and solubility.

Consequently, the stickiness was controlled to ensure sufficient adherence to the bone tissue surface but minimal adherence to the surgical gloves. This control avoids the inconvenience of adding pregelatinized starch to improve the molding texture, as proposed by Suwanprateeb et al. [16].

In addition, OSSTOP maintained hemostasis within a bleeding femur defect in a rat model, ultimately allowing uninhibited bone regeneration. When applying a bone wax to an affected area in clinical environments, the important factors are usability/spreadability, hemostatic performance, hemostasis maintenance, and absorption/decomposition of the bone wax within the body. These properties of the commercial and proposed bone waxes were compared through animal experiments.

The animal experiments revealed higher usability/spreadability and greater hemostatic performances of OSSTOP than of the commercially available OSTENE and NOVOSEAL. Five of 8 individuals treated with NOVOSEAL yielded a “Grade 2” usability/spreadability rating. In the OSTENE and OSSTOP groups, the usability/spreadability ratings improved to “Grade 3” for 6/8 individuals and all 8 individuals, respectively, confirming that OSSTOP improves the tissue adhesion, spreadability, and user convenience. During the 5-min hemostatic performance evaluation, NOVOSEAL exhibited various degrees of hemostatic performance (“Grade 0” to “Grade 4”), whereas OSTENE and OSSTOP exhibited “Grade 4” hemostatic performance in 7/8 cases and in all 8 cases, respectively. During 60 min of evaluation, the NOVOSEAL, OSTENE, and OSSTOP maintained the hemostatic state for 18.6, 22.5, and 56.8 min on average, respectively. That is, the average hemostatic maintenance time was approximately 3.0 and 2.5 times longer in the OSSTOP group than in the NOVOSEAL and OSTENE groups, respectively. In conclusion, the usability/spreadability and tissue adhesion performances of the test substance OSSTOP exceed those of the control groups OSTENE and NOVOSEAL, leading to excellent results in the 5-min evaluation of hemostatic performance and the 60-min evaluation of hemostatic maintenance time. In addition, OSSTOP and OSTENE were rated “Grade 2” in the absorption/degradability evaluation, denoting that more than 75% of the bone wax was absorbed/degraded at 1 d post-application. OSSTOP and OSTENE were absorbed/degraded to undetectable levels (naked eye) at 3 d post-application, improving their rating to “Grade 3”. During the histopathological evaluations of inflammatory and tissue-regeneration responses, OSSTOP, OSTENE, and the negative control group exhibited similar degrees of inflammatory responses at 1 and 3 d post-application. All 3 groups also exhibited similar granulation tissue formations and bone-regeneration responses at 7 and 14 d post-application. Similar results were reported by Suwanprateeb et al. and numerous other studies [16,22–25].

Furthermore, in the 13-week long-term observation study of femoral implantation in the rabbit model, no animal deaths occurred in any test group throughout the study period. Additionally, no abnormal symptoms related to the implantation of the test materials were observed in any group, including the control group, confirming the long-term safety of the material.

The developed OSSTOP absorbable wax can effectively adhere to the surfaces of bleeding bone and bone-amputation sites, exerting a physical hemostatic effect and higher usability/spreadability, hemostatic performance, and hemostasis maintenance than the existing commercialized bone waxes. In addition, a post-transplantation histopathological evaluation confirmed an absence of granulation tissue formation and a bone-regeneration reaction, confirming the potential of OSSTOP in clinical applications.

Additionally, OSSTOP has been approved by the Korean Ministry of Food and Drug Safety (MFDS) for human application and is manufactured in medical device Good Manufacturing Practice (GMP) facilities. The synthesis and molding processes can be integrated into an automated production system, which is expected to contribute to the medical industry.

### Conclusion

Through thermal synthesis of mixed liquid and powder poloxamers, we developed an absorbable bone wax (OSSTOP) with an improved molding texture and strong mechanical properties. During animal testing experiments simulating a clinical use environment, OSSTOP demonstrated higher hemostasis performance (usability/spreadability, hemostatic performance, and hemostasis maintenance) than the existing commercialized bone wax. In addition, a post-transplantation histopathological evaluation confirmed that OSSTOP does not inhibit the bone-regeneration reaction, confirming that it can adequately treat bone bleeding and amputation. OSSTOP is expected to increase user satisfaction and achieve rapid hemostasis of the affected area in clinical use environments, although its clinical applicability must be evaluated in further work.

## Data Availability

The datasets used and analyzed during the current study are available from the corresponding author on reasonable request.
