# Supplementary material for: Physicochemical Properties and Efficacy of Poloxamer Bone Wax on Hemostasis at the Bone-Amputation Site
Source: Biomater Res. 2025 Apr 15;29:0191. doi: 10.34133/bmr.0191 (PMC11997309; doi:10.34133/bmr.0191)
Supplement: Supplementary 1 — Figs. S1 and S2 Tables S1 and S2 [file bmr.0191.f1.docx]

**
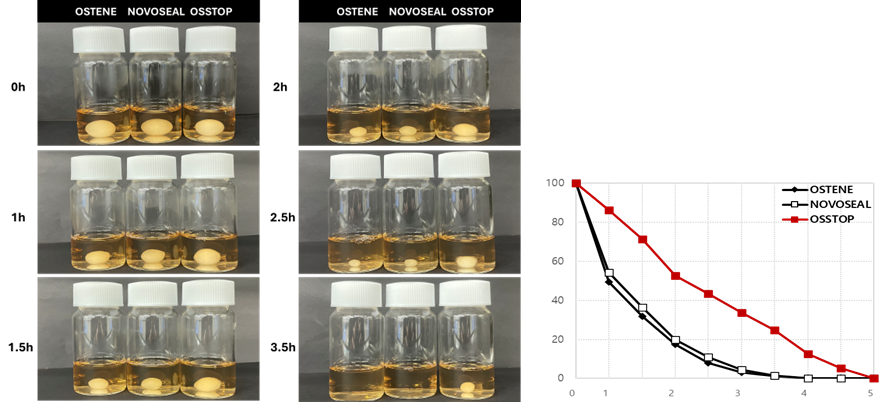
**

**Supplementary Figure 1.** Solubility comparison of OSSTOP and commercialized bone wax products in FBS solution.

| **Group** | | **Sex** | **Animal No.** |
| --- | --- | --- | --- |
| G1 | OSTENE  (Control) | Male | M1~M4 |
|  |  | Female | F1~F4 |
| G2 | OSSTOP  (Test material) | Male | M5~M8 |
|  |  | Female | F5~F8 |

A


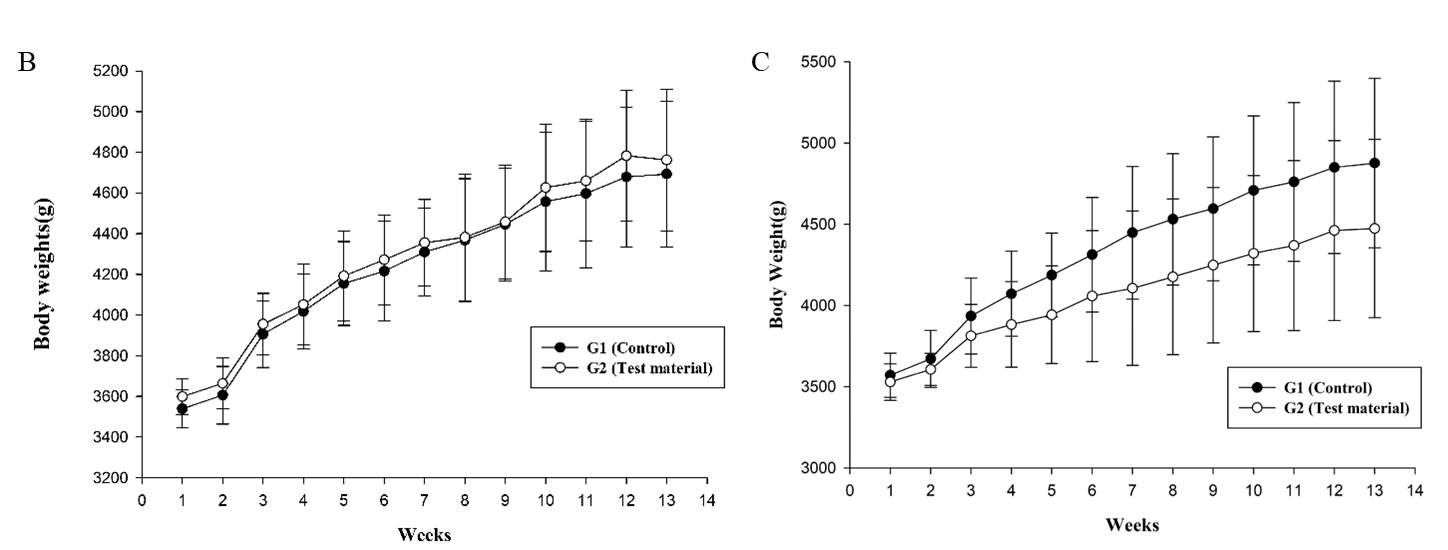


**Supplementary Figure 2.** Composition of test of transplant during 13 weeks. (A) Composition of test group with individual identification change in body weight of (B)male and (C)female rabbit

**Supplementary Table 1**. Scoring of Histological observation evaluation and Microscopic findings of implantation site in male/female rabbits

| Category | Score | | | | |
| --- | --- | --- | --- | --- | --- |
|  | 0 | 1 | 2 | 3 | 4 |
| Polymorphonuclear cells | 0 | Rare, 1-5/phf* | 5-10/phf | Heavy infiltrate | Packed |
| Lymphocytes | 0 | Rare, 1-5/phf | 5-10/phf | Heavy infiltrate | Packed |
| Plasma cells | 0 | Rare,1-5/phf | 5-10/phf | Heavy infiltrate | Packed |
| Macrophages | 0 | Rare, 1-5/phf | 5-10/phf | Heavy infiltrate | Packed |
| Giant cells Necrosis | 0 | Rare, 1-2/phf | 3-5/phf | Heavy infiltrate | Sheets |
| Neovascularisation | 0 | Minimal  capillary  proliferation,  focal, 1-3  buds | Groups of 4-7  capillaries with  supporting  fibroblastick  structures | Broad band of capillaries with supporting structures | Extensive band  of capillaries  with supporting  fibroblastic  structures |
| Fibrosis | 0 | Narrow band | Moderately  thick band | Thick band | Extensive band |
| Fatty infiltrate | 0 | Minimal amount of fat  associated with fibrosis | Several layers of fat and fibrosis | Elongated and broad  accumulation of fat cells about  the implant site | Extensive fat completely surrounding the implant |

* : phf : per high powered (400×) field

| Implantation | Category | Animal Number | | | | | | | | | | | Animal Number | | | | | | | | | |
| --- | --- | --- | --- | --- | --- | --- | --- | --- | --- | --- | --- | --- | --- | --- | --- | --- | --- | --- | --- | --- | --- | --- |
|  |  | M1-T1^a^ | M1-T2 | M1-T3 | M2-T1 | M2-T2 | M2-T3 | M3-T1 | M3-T2 | M4-T1 | M4-T2 | F1-T1^a^ | | F1-T2 | F1-T3 | F2-T1 | F2-T2 | F2-T3 | F3-T1 | F3-T2 | F4-T1 | F4-T2 |
| G1 | Inflammation | 0 | 0 | 0 | 0 | 0 | 0 | 0 | 0 | 0 | 0 | 0 | | 0 | 0 | 0 | 0 | 0 | 0 | 0 | 0 | 0 |
|  | Polymorphonuclear leukocyte |  |  |  |  |  |  |  |  |  |  |  |  |  |  |  |  |  |  |  |  |  |
|  | Lymphocytes | 0 | 0 | 0 | 0 | 0 | 0 | 0 | 0 | 0 | 0 | 0 | | 0 | 0 | 0 | 0 | 0 | 0 | 0 | 0 | 0 |
|  | Plasma cells | 0 | 0 | 0 | 0 | 0 | 0 | 0 | 0 | 0 | 0 | 0 | | 0 | 0 | 0 | 0 | 0 | 0 | 0 | 0 | 0 |
|  | Macrophages | 0 | 0 | 0 | 0 | 0 | 0 | 0 | 0 | 0 | 0 | 0 | | 0 | 0 | 0 | 0 | 0 | 0 | 0 | 0 | 0 |
|  | Giant cells | 0 | 0 | 0 | 0 | 0 | 0 | 0 | 0 | 0 | 0 | 0 | | 0 | 0 | 0 | 0 | 0 | 0 | 0 | 0 | 0 |
|  | Necrosis | 0 | 0 | 0 | 0 | 0 | 0 | 0 | 0 | 0 | 0 | 0 | | 0 | 0 | 0 | 0 | 0 | 0 | 0 | 0 | 0 |
|  | Sub-Total (×2) | 0 | 0 | 0 | 0 | 0 | 0 | 0 | 0 | 0 | 0 | 0 | | 0 | 0 | 0 | 0 | 0 | 0 | 0 | 0 | 0 |
|  | Neovascularisation | 1 | 0 | 1 | 1 | 1 | 0 | 1 | 1 | 0 | 0 | 1 | | 0 | 0 | 0 | 0 | 0 | 1 | 1 | 1 | 1 |
|  | Fibrosis | 0 | 0 | 0 | 0 | 0 | 0 | 0 | 0 | 0 | 0 | 0 | | 0 | 0 | 0 | 0 | 0 | 0 | 0 | 0 | 0 |
|  | Fatty infiltrate | 0 | 0 | 0 | 0 | 0 | 0 | 0 | 0 | 0 | 0 | 0 | | 0 | 0 | 0 | 0 | 0 | 0 | 0 | 0 | 0 |
|  | Sub-Total | 1 | 0 | 1 | 1 | 1 | 0 | 1 | 1 | 0 | 0 | 1 | | 0 | 0 | 0 | 0 | 0 | 1 | 1 | 1 | 1 |
|  | Total | 1 | 0 | 1 | 1 | 1 | 0 | 1 | 1 | 0 | 0 | 1 | | 0 | 0 | 0 | 0 | 0 | 1 | 1 | 1 | 1 |
|  | Group Total | 6 | | | | | | | | | | | 5 | | | | | | | | | |
|  | Traumatic necrosis | 0 | 0 | 0 | 0 | 0 | 0 | 0 | 0 | 0 | 0 | 0 | | 0 | 0 | 0 | 0 | 0 | 0 | 0 | 0 | 0 |
|  | Foreign debris | 0 | 0 | 0 | 0 | 0 | 0 | 0 | 0 | 0 | 0 | 0 | | 0 | 0 | 0 | 0 | 0 | 0 | 0 | 0 | 0 |
|  | No. sites examined | 10 | | | | | | | | | | | 10 | | | | | | | | | |
| G2 | Category | Animal Number | | | | | | | | | | | Animal Number | | | | | | | | | |
|  |  | M5-T1^a^ | M5-T2 | M5-T3 | M6-T1 | M6-T2 | M6-T3 | M7-T1 | M7-T2 | M8-T1 | M8-T2 | F5-T1^a^ | | F5-T2 | F5-T3 | F6-T1 | F6-T2 | F6-T3 | F7-T1 | F7-T2 | F8-T1 | F8-T2 |
|  | Inflammation | 0 | 0 | 0 | 0 | 0 | 0 | 0 | 0 | 0 | 0 | 0 | | 0 | 0 | 0 | 0 | 0 | 0 | 0 | 0 | 0 |
|  | Polymorphonuclear leukocyte |  |  |  |  |  |  |  |  |  |  |  |  |  |  |  |  |  |  |  |  |  |
|  | Lymphocytes | 0 | 0 | 0 | 0 | 0 | 0 | 0 | 0 | 0 | 0 | 0 | | 0 | 0 | 0 | 0 | 0 | 0 | 0 | 0 | 0 |
|  | Plasma cells | 0 | 0 | 0 | 0 | 0 | 0 | 0 | 0 | 0 | 0 | 0 | | 0 | 0 | 0 | 0 | 0 | 0 | 0 | 0 | 0 |
|  | Macrophages | 1 | 0 | 0 | 0 | 0 | 0 | 0 | 0 | 0 | 0 | 0 | | 0 | 0 | 0 | 0 | 0 | 0 | 0 | 0 | 0 |
|  | Giant cells | 0 | 0 | 0 | 0 | 0 | 0 | 0 | 0 | 0 | 0 | 0 | | 0 | 0 | 0 | 0 | 0 | 0 | 0 | 0 | 0 |
|  | Necrosis | 0 | 0 | 0 | 0 | 0 | 0 | 0 | 0 | 0 | 0 | 0 | | 0 | 0 | 0 | 0 | 0 | 0 | 0 | 0 | 0 |
|  | Sub-Total (×2) | 2 | 0 | 0 | 0 | 0 | 0 | 0 | 0 | 0 | 0 | 0 | | 0 | 0 | 0 | 0 | 0 | 0 | 0 | 0 | 0 |
|  | Neovascularisation | 2 | 0 | 0 | 0 | 0 | 0 | 0 | 2 | 0 | 0 | 0 | | 1 | 1 | 0 | 0 | 2 | 0 | 0 | 0 | 0 |
|  | Fibrosis | 0 | 0 | 0 | 0 | 0 | 0 | 0 | 0 | 0 | 0 | 0 | | 0 | 0 | 0 | 0 | 0 | 0 | 0 | 0 | 0 |
|  | Fatty infiltrate | 0 | 0 | 0 | 0 | 0 | 0 | 0 | 0 | 0 | 0 | 0 | | 0 | 0 | 0 | 0 | 0 | 0 | 0 | 0 | 0 |
|  | Sub-Total | 2 | 0 | 0 | 0 | 0 | 0 | 0 | 2 | 0 | 0 | 0 | | 1 | 1 | 0 | 0 | 2 | 0 | 0 | 0 | 0 |
|  | Total | 4 | 0 | 0 | 0 | 0 | 0 | 0 | 2 | 0 | 0 | 0 | | 1 | 1 | 0 | 0 | 2 | 0 | 0 | 0 | 0 |
|  | Group Total | 6 | | | | | | | | | | | 4 | | | | | | | | | |
|  | Traumatic necrosis | 0 | 0 | 0 | 0 | 0 | 0 | 0 | 0 | 0 | 0 | 0 | | 0 | 0 | 0 | 0 | 0 | 0 | 0 | 0 | 0 |
|  | Foreign debris | 0 | 0 | 0 | 0 | 0 | 0 | 0 | 0 | 0 | 0 | 0 | | 0 | 0 | 0 | 0 | 0 | 0 | 0 | 0 | 0 |
|  | No. sites examined | 10 | | | | | | | | | | | 10 | | | | | | | | | |
| Average^b^ | | 0 | | | | | | | | | | | 0 | | | | | | | | | |

^a^ : Tissue implantation site

^b^ : Average =Test (Group Total Score / # site) - Control (Group Total Score / # site)

- **Bioreactivity Rating = ' 0.0 ' [Average (Test – Control)]**

**Supplementary Table 2.** Results of the hematological and blood biochemical analyses (A) Blood Chemical values, (B)Hematological values, (C) Histopathological findings

**(A) Blood Chemical values**

| **Sex** | Group | | TP | ALB | A/G | T-BIL | ALP | ATS | ALT | CREA | BUN | T-CHO | TG | GLU | CA | IP | GGT | CK | Na | K | Cl |
| --- | --- | --- | --- | --- | --- | --- | --- | --- | --- | --- | --- | --- | --- | --- | --- | --- | --- | --- | --- | --- | --- |
|  |  |  | (g/dl) | (g/dl) | - | (mg/dl) | (U/L) | (U/L) | (U/L) | (mg/dl) | (mg/dl) | (mg/dl) | (mg/dl) | (mg/dl) | (mg/dl) | (mg/dl) | (IU/L) | (IU/L) | (mmol/L) | (mmol/L) | (mmol/L) |
| Male | G1 | Mean | 6.1 | 3.6 | 1.5 | 0.00 | 87 | 30 | 46 | 1.56 | 21.9 | 18 | 19 | 126 | 12.9 | 5.9 | 5.16 | 1607 | 145.7 | 4.72 | 105.3 |
|  |  | S.D. | 0.4 | 0.1 | 0.1 | 0.00 | 21 | 13 | 11 | 0.25 | 2.5 | 3 | 9 | 10 | 0.3 | 0.6 | 1.85 | 1121 | 1.2 | 0.49 | 1.2 |
|  |  | N | 4 | 4 | 4 | 4 | 4 | 4 | 4 | 4 | 4 | 4 | 4 | 4 | 4 | 4 | 4 | 4 | 4 | 4 | 4 |
|  | G2 | Mean | 6.0 | 3.6 | 1.5 | 0.00 | 90 | 25 | 54 | 1.33 | 19.7 | 18 | 18 | 132 | 12.5 | 5.3 | 5.32 | 1179 | 143.8 | 4.02 | 105.7 |
|  |  | S.D. | 0.3 | 0.1 | 0.2 | 0.00 | 13 | 5 | 9 | 0.18 | 2.7 | 4 | 2 | 9 | 0.4 | 1.3 | 1.62 | 246 | 1.4 | 0.52 | 1.4 |
|  |  | N | 4 | 4 | 4 | 4 | 4 | 4 | 4 | 4 | 4 | 4 | 4 | 4 | 4 | 4 | 4 | 4 | 4 | 4 | 4 |
| Female | G1 | Mean | 6.0 | 3.5 | 1.4 | 0.00 | 160 | 19 | 53 | 1.54 | 28.8 | 39 | 12 | 122 | 13.3 | 4.9 | 7.32 | 676 | 144.1 | 4.27 | 109.2 |
|  |  | S.D. | 0.2 | 0.1 | 0.1 | 0.00 | 81 | 1 | 9 | 0.19 | 1.1 | 12 | 3 | 3 | 0.2 | 0.5 | 1.39 | 245 | 0.7 | 0.21 | 0.8 |
|  |  | N | 4 | 4 | 4 | 4 | 4 | 4 | 4 | 4 | 4 | 4 | 4 | 4 | 4 | 4 | 4 | 4 | 4 | 4 | 4 |
|  | G2 | Mean | 5.6* | 3.4 | 1.5 | 0.00 | 95 | 22 | 47 | 1.53 | 29.7 | 40 | 15 | 127 | 12.9* | 4.8 | 7.10 | 660 | 142.6* | 4.06 | 108.1 |
|  |  | S.D. | 0.1 | 0.1 | 0.2 | 0.00 | 25 | 5 | 13 | 0.17 | 3.0 | 4 | 2 | 8 | 0.3 | 0.1 | 1.66 | 113 | 0.9 | 0.09 | 0.8 |
|  |  | N | 4 | 4 | 4 | 4 | 4 | 4 | 4 | 4 | 4 | 4 | 4 | 4 | 4 | 4 | 4 | 4 | 4 | 4 | 4 |

**(B)Hematological values**

| Sex | Group | | WBC | WBC Differential Counting(%) | | | | | RBC | Hb | HCT | MCV | MCH | MCHC | Retic | PLT | PT | APTT |
| --- | --- | --- | --- | --- | --- | --- | --- | --- | --- | --- | --- | --- | --- | --- | --- | --- | --- | --- |
|  |  |  | (K/ul) | NE | LY | MO | EO | BA | (M/ul) | (g/㎗) | (%) | (fL) | (pg) | (g/㎗) | (%) | (K/ul) | (sec) | (sec) |
| Meal | G1 | Mean | 5.79 | 42.6 | 50.2 | 1.2 | 2.5 | 3.3 | 6.54 | 13.8 | 42.5 | 65.1 | 21.2 | 32.6 | 2.76 | 394 | 7.5 | 22.1 |
|  |  | S.D. | 0.98 | 8.7 | 6.5 | 0.4 | 0.7 | 1.7 | 0.38 | 0.6 | 1.9 | 2.3 | 0.7 | 0.4 | 0.45 | 124 | 0.6 | 5.5 |
|  |  | N | 4 | 4 | 4 | 4 | 4 | 4 | 4 | 4 | 4 | 4 | 4 | 4 | 4 | 4 | 4 | 4 |
|  | G2 | Mean | 6.30 | 38.4 | 51.8 | 2.1 | 2.3 | 4.9 | 6.56 | 14.5 | 44.0 | 67.1 | 22.1 | 33.0 | 3.03 | 326 | 7.4 | 18.9 |
|  |  | S.D. | 2.17 | 17.9 | 16.4 | 1.0 | 0.9 | 2.5 | 0.62 | 1.2 | 3.0 | 2.2 | 0.6 | 0.6 | 0.36 | 41 | 0.5 | 3.4 |
|  |  | N | 4 | 4 | 4 | 4 | 4 | 4 | 4 | 4 | 4 | 4 | 4 | 4 | 4 | 4 | 4 | 4 |
| Female | G1 | Mean | 5.55 | 40.7 | 51.0 | 1.5 | 2.1 | 3.9 | 6.42 | 14.1 | 42.5 | 66.3 | 22.0 | 33.1 | 2.67 | 432 | 7.0 | 24.3 |
|  |  | S.D. | 0.93 | 9.7 | 9.1 | 0.6 | 0.4 | 1.1 | 0.23 | 0.3 | 1.3 | 3.8 | 1.2 | 0.3 | 0.72 | 114 | 0.0 | 4.7 |
|  |  | N | 4 | 4 | 4 | 4 | 4 | 4 | 4 | 4 | 4 | 4 | 4 | 4 | 4 | 4 | 4 | 4 |
|  | G2 | Mean | 3.91* | 42.8 | 47.7 | 1.6 | 2.5 | 5.2 | 6.12 | 13.0* | 40.0* | 65.4 | 21.3 | 32.5* | 2.69 | 403 | 7.1 | 18.6 |
|  |  | S.D. | 0.83 | 11.5 | 13.9 | 0.5 | 0.8 | 1.8 | 0.23 | 0.6 | 1.5 | 0.2 | 0.2 | 0.4 | 0.21 | 128 | 0.3 | 4.0 |
|  |  | N | 4 | 4 | 4 | 4 | 4 | 4 | 4 | 4 | 4 | 4 | 4 | 4 | 4 | 4 | 4 | 4 |

N : Animal Numbers, WBC : Total leucocyte count, NE: Neutrophils, LY: Lymphocytes, MO: Monocytes, EO: Eosinophils, BA: Basophils, RBC : Total erythrocyte count, Hb: Hemoglobin concentration, HCT : Hematocrit, MCV : Mean cell volume, MCH : Mean cell hemoglobin, MCHC : Mean cell hemoglobin Concentration, Retic : Reticulocyte, PLT : Platelet, PT : Prothrombin time, APTT : Activated partial thromboplastin time.

Significant differences as compared with control : *P<0.05

**(C) Histopathological findings**

| Time of extraction | Organs | No. of animal examined | Histopathological findings | Male | | Female | |
| --- | --- | --- | --- | --- | --- | --- | --- |
|  |  |  |  | G1 | G2 | G1 | G2 |
| 13 weeks | Liver / Gall  bladder | 4 | No abnormalities detected / No abnormalities detected | 3 | 4 | 3 | 4 |
|  |  |  | Hepatocyte vacuolation minimal multifocal /  No abnormalities detected | 1 | - | - | - |
|  |  |  | Inflammatory cell foci minimal multifocal /  No abnormalities detected | - | - | 1 | - |
|  | Kidney | 4 | No abnormalities detected 4 3 | 4 | 3 | 4 | 3 |
|  |  |  | Scar, cortical/present | - | 1 | - | - |
|  |  |  | Inflammatory cell infiltration, interstitial minimal multifocal | - | - | - | 1 |
|  | Adrenal gland | 4 | No abnormalities detected | 3 | 4 | 4 | 4 |
|  |  |  | Vacuolation, cortical minimal diffuse | 1 | - | - | - |
|  | Heart | 4 | No abnormalities detected | 4 | 4 | 4 | 3 |
|  |  |  | Inflammatory cell infiltration, myocardial slight multifocal | - | - | 2- | 1 |
|  | Lung | 4 | No abnormalities detected | 3 | 2 | 2 | 4 |
|  |  |  | Macrophage infiltration, alveolar minimal multifocal | 1 | 2 | 2 | - |
|  | Brain  (Cerebrum) | 4 | No abnormalities detected | 4 | 4 | 4 | 4 |
|  | Brain  (Cerebellum) | 4 | No abnormalities detected | 4 | 4 | 4 | 4 |
|  | Brain  (Pituitary gland) | 4 | No abnormalities detected | 4 | 3 | 4 | 4 |
|  |  |  | Cyst, part intermediate/present | - | 1 | - | - |
|  | Spinal cord | 4 | No abnormalities detected | 4 | 4 | 4 | 4 |
|  | Seminal vesicle | 4 | No abnormalities detected | 4 | 4 | - | - |
|  | Spleen | 4 | No abnormalities detected | 3 | 2 | 4 | 4 |
|  |  |  | Pigment minimal multifocal | 1 | 2 | - | - |
|  | Testis | 4 | No abnormalities detected | 4 | 4 | - | - |
|  | Ovary | 4 | No abnormalities detected | - | - | 4 | 2 |
|  |  |  | Hemorrhagic follicle/present | - | - | - | 2 |
|  | Epididymis | 4 | No abnormalities detected | 4 | 4 | - | - |
|  | Uterus | 4 | No abnormalities detected | - | - | 4 | 4 |
|  | Prostate | 4 | No abnormalities detected | 4 | 4 | - | - |
|  | Vagina | 4 | No abnormalities detected | - | - | 4 | 4 |
|  | Trachea | 4 | No abnormalities detected | 4 | 4 | 4 | 4 |
|  | Esophagus | 4 | No abnormalities detected | 4 | 4 | 4 | 4 |
|  | Thymus | 4 | No abnormalities detected | 4 | 4 | 4 | 4 |
|  | Thyroid /  parathyroid | 4 | No abnormalities detected / No abnormalities detected | 4 | 4 | 4 | 4 |
|  | Stomach | 4 | No abnormalities detected | 4 | 4 | 4 | 4 |
|  | Duodenum | 4 | No abnormalities detected | 4 | 4 | 4 | 4 |
|  | Urinary Bladder | 4 | No abnormalities detected | 2 | 2 | 3 | 2 |
|  |  |  | Concretion minimal multifocal | 2 | 2 | 1 | 2 |
|  | Intestine | 4 | No abnormalities detected | 4 | 4 | 4 | 4 |
|  | Eye/Harderian  gland | 4 | No abnormalities detected/ No abnormalities detected | 4 | 4 | 4 | 4 |
|  | Skin | 4 | No abnormalities detected | 4 | 4 | 4 | 4 |
|  | Submandibular  gland | 4 | No abnormalities detected | 4 | 4 | 4 | 4 |
|  | Skeletal muscle  /  Sciatic nerve | 4 | No abnormalities detected | 4 | 4 | 4 | 4 |
|  | Pancreas | 4 | No abnormalities detected | 4 | 4 | 4 | 4 |
|  | Intestinal  Lymphnode | 4 | No abnormalities detected | 4 | 4 | 4 | 4 |
|  | Femur | 4 | No abnormalities detected | 4 | 4 | 4 | 4 |
|  | Sternum | 4 | No abnormalities detected | 4 | 4 | 4 | 4 |
